# *Prunus cerasoides* Extract and Its Component Compounds Upregulate Neuronal Neuroglobin Levels, Mediate Antioxidant Effects, and Ameliorate Functional Losses in the Mouse Model of Cerebral Ischemia

**DOI:** 10.3390/antiox11010099

**Published:** 2021-12-30

**Authors:** So-Dam Kim, Minha Kim, Hong-Hua Wu, Byung Kwan Jin, Myung-Shin Jeon, Yun Seon Song

**Affiliations:** 1 Department of Pharmacology, College of Pharmacy, Sookmyung Women’s University, Seoul 04310, Korea; 1531313@sookmyung.ac.kr; 2 Translational Research Center, Department of Molecular Biomedicine, IRIMS and College of Medicine, Inha University, Incheon 22332, Korea; black359@naver.com (M.K.); msjeon@inha.ac.kr (M.-S.J.); 3 State Key Laboratory of Component-Based Chinese Medicine, Institute of Traditional Chinese Medicine, Tianjin University of Traditional Chinese Medicine, 10 Poyanghu Road, West Area, Tuanbo New Town, Jinghai District, Tianjin 301617, China; wuhonghua2011@tjutcm.edu.cn; 4 Department of Biochemistry & Molecular Biology, School of Medicine, Kyung Hee University, Seoul 02447, Korea; bkjin@khu.ac.kr; 5 Program in Biomedical Science and Engineering, Graduate School, Inha University, Incheon 22332, Korea

**Keywords:** *Prunus cerasoides*, neuroglobin, stroke, antioxidant, apoptosis, neuroprotection

## Abstract

*Prunus cerasoides* (PC) has been reported to have antimicrobial and anti-inflammatory properties, but its potential as a neuroprotective agent in a mouse model of cerebral ischemia has not been explored. Considering neuroglobin (Ngb), an endogenous neuroprotective factor, as a novel approach to neuroprotection, in this study, Ngb promoter activity, Ngb expression changes, and antioxidant protection by PC extract (PCE) and PC component compounds (PCCs) were analyzed in oxygen–glucose deprivation (OGD)-treated neurons. In vivo analysis involved transient middle cerebral artery occlusion (tMCAO) in mice with pre- and post-treatment exposure to PCE. Following ischemic stroke induction, neurological behavior scores were obtained, and cellular function-related signals were evaluated in the ischemic infarct areas. In addition to PCE, certain component compounds from PCE also significantly increased Ngb levels and attenuated the intracellular ROS production and cytotoxicity seen with OGD in primary neurons. Administration of PCE reduced the infarct volume and improved neurological deficit scores in ischemic stroke mice compared with the vehicle treatment. Increased Ngb levels in infarct penumbra with PCE treatment were also accompanied by decreased markers of apoptosis (activated p38 and cleaved caspase-3). Our findings point to the benefits of Ngb-mediated neuroprotection via PCE and its antioxidant activity in an ischemic stroke model.

## 1. Introduction

Ischemic stroke, as a vascular event, is caused by the blockage of an artery that supplies oxygen and nutrients to the brain, leading to the damage and death of brain cells [1,2,3]. It also leads to a secondary impairment called ischemia–reperfusion (I/R) injury, manifested as additional damage to the affected area once the blood flow is restored; I/R results in a further ischemic cascade, leading to a neurodegenerative-like condition [4,5]. Part of cerebral I/R injury is caused by the formation of reactive oxygen and nitrogen species (ROS and RNS) that exacerbate the condition of affected tissues over time. Anatomically, blood flow reduction is most severe at the center of the ischemic territory, which is called the ischemic core, and this results in rapid cell death. However, at the periphery of the ischemic territory, called the penumbra, cerebral blood flow is sufficient to keep neurons alive [6]. Pathological progression leads to the growth of the center ischemic region into the penumbral region and expansion of the injury to the entire ischemic area. To this end, therapeutic approaches are required to identify the potentially salvageable penumbra and to promote survival-related mechanisms targeting the penumbra.

Neuroglobin (Ngb), a member of the globin family, was discovered in 2000 [7] and is a highly conserved protein. It has a high affinity for oxygen and is mainly expressed in the neurons of the central and peripheral nervous system (CNS and PNS), as well as in the retina [8,9,10]. Ngb exerts a critical antiapoptotic function in cells exposed to accumulating oxidative stress, as it acts as an oxygen sensor and ROS scavenger [11,12,13]. In the mitochondrial pathway of apoptosis, Ngb inhibits cytochrome C release, directly interfering with the apoptotic pathway and affecting various types of upstream stressor signals in the neuron [14]. Thus, a promising neuroprotective strategy may be to increase Ngb levels in the penumbra area as it is experiencing oxidative stress.

*Prunus cerasoides* (PC) has been used as a traditional medicine for wound healing, backaches, and sprains [15,16], and its phytochemical profile shows the presence of bioactive constituents, including terpenoids, flavonoids, and phenolics [17,18,19,20]. Previous studies have focused on the antimicrobial, anti-inflammatory, and antiplasmodial activities of PC [21,22,23,24]. Earlier, we reported on the isolation of single components of PC extract (PCE), and their chemical structures were identified [25]. It has also been observed that PCE and its bioactive components have estrogenic activities in vitro and in vivo in relevant preclinical models. Despite previous studies on the beneficial properties of PC, the potential benefit of PCE in neuroprotection has not been studied. As flavonoids promote the expression of Ngb [26], which is closely associated with neurological disease progression, flavonoids and isoflavonoids in PC could also lead to increased Ngb expression in treated cells and animal models. Therefore, this study investigated whether PCE upregulates Ngb expression in cell-based systems and whether PCE leads to protection against cerebral ischemic damage following tMCAO in mice.

## 2. Materials and Methods

### 2.1. Prunus cerasoides (PC) Sourcing and Characterization

Fresh bark, xylem, stems, and leaves of PC were obtained from the Dali Bai autonomous prefecture of Yunnan province, China; they were identified by Dr. Sang Woo Lee of Korea Research Institute of Bioscience and Biotechnology (KRIBB, Daejeon, Korea) in 2012 and were analyzed by Dr. Sei-Ryang Oh (KRIBB, Ochang, Korea). A voucher specimen (accession number 0082450) of the retained material has been stored at the herbarium of KRIBB (Daejeon). The PC materials were first processed and then analytically isolated and characterized. Briefly, for processing, collected PC stems (PCS, 640 g) and PC leaves (PCL, 350 g) were first cleaned properly, dried, and then ultrasonically extracted twice by 95% ethanol to obtain the PCS (45 g) and PCL extracts (35 g), respectively. Eighteen compounds from the extracts were described in detail in our previous report [25]. In Table 1, 18 compounds present in PCE with their structure/formula are listed.

### 2.2. Cell Culture

Human neuroblastoma (SKNSH) and mouse neuroblastoma (N2a) cells were transfected with Ngb promoter-reporter plasmids as well as the plasmid pRL-TK, which encodes *Renilla* luciferase for transfection efficiency, and these were kindly provided by Dr. Xiaoying Wang (Tulane University School of Medicine, New Orleans, LA, USA) [27]. In an incubator with humidified atmosphere and 5% CO_2_, SKNSH and N2a cells were cultured in Dulbecco’s modified Eagle’s medium (DMEM) (Sigma-Aldrich, St. Louis, MO, USA) supplemented with 1% antibiotic–antimycotic solution (Gibco/Thermo Fisher, Carlsbad, CA, USA) and 10% fetal bovine serum (FBS) (Gibco/Thermo Fisher) at 37 °C.

### 2.3. Materials and Reagents

Most of the reagents and chemicals were purchased from Gibco/Thermo Fisher, Welgene (Gyeongsan, Korea), and Sigma Aldrich. All materials employed in this research were of research grade, suitable for cell culture. Unless specified, each of the chemicals for analysis and the assays were first dissolved in dimethyl sulfoxide (DMSO) before being used. The final DMSO solvent concentration in the culture medium, however, did not exceed 0.5% (vol/vol) when used for a biological assay.

### 2.4. Animals

All animal studies were in accordance with the current guidelines for animal welfare of Sookmyung Women’s University. The animal protocols were first reviewed and then approved by the Institutional Animal Care and Use Committee (IACUC) of the university (approval number SMWU-IACUC-1911-022). Eight- to ten-week-old female C57BL/6 mice (20–25 g) were purchased from Saeronbio Inc. (Uiwang, Korea). The animals were housed in a purpose-built facility with a controlled environment and were maintained in isolation at a temperature and relative humidity of 24 ± 2 °C and 50%, respectively. The lighting was artificial with a 12 h light/dark cycle, and the animals were provided with sterile water and food ad libitum.

**Scheme 1:**
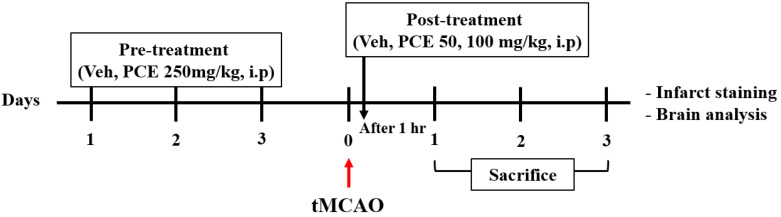
The timeline of experiments. Ischemic stroke was induced in all mice. For preadministration, mice were randomly intraperitoneally administered vehicle or PCE (250 mg/kg) for 3 consecutive days prior to tMCAO. For postadministration, mice were intraperitoneally administered vehicle or PCE (50, 100 mg/kg) once after tMCAO.

To examine the effect of drug pretreatment, the animals were randomly divided into 5 groups and received the following treatment (Scheme 1): the sham and vehicle-tMCAO groups were administered 1% carboxymethyl cellulose (CMC) in 0.9% NaCl (i.p), and the PCE group was administered 1% CMC in 0.9% NaCl containing PCE (250 mg/kg, i.p) for 3 consecutive days before ischemic stroke. Moreover, the treatment to examine the effect of the post-treatment was as follows: the sham and vehicle-tMCAO groups were administered 1% CMC in 0.9% NaCl (i.p), and the PCE group was administered 1% CMC in 0.9% NaCl containing PCE (50 or 100 mg/kg, i.p). In the case of post-treatment, it was intraperitoneally injected once 1 h after ischemic stroke. At the end of the experiment, all animals were euthanized, followed by the collection of brain samples.

### 2.5. Transient Middle Cerebral Artery Occlusion (tMCAO)

The tMCAO stroke model was induced via occlusion of the middle cerebral artery using a 6-0 nylon monofilament suture (Doccol, Sharon, MA, USA). Briefly, the mice were anesthetized using 1.6–2% isoflurane (Vspharm, Korea) in oxygen/nitrous oxide (1:2) using a face mask. The mice were placed on a heat pad, and their cerebral blood flow (CBF) was monitored with a laser-Doppler probe (PeriFlux System 5000 with PF 5010) (Perimed, Järfälla, Sweden) throughout the procedure. The mice were subjected to 45 min of tMCAO as described previously with minor modifications [28]. This involved a suture with a round, coated tip (Cat# 602256PK10, Cat# 602356PK10; Doccol Corporation, Sharon, MA, USA) being inserted into the external carotid artery (ECA) lumen, which was then gently advanced into the internal carotid artery (ICA) lumen to block the MCA blood flow and was left there for 45 min, until reperfusion. In sham-operated mice, after incision of the skin and exposure of blood vessels (as in tMCAO), a knot was made in the common carotid artery (CCA). After 10 min, the knot was removed, and the skin was sutured. After surgery, the mice were euthanized at various time points. The exclusion criteria were as follows: (1) death during surgery or anesthesia problems; (2) a reduction in CBF to less than 20–30% of baseline; (3) death before sampling; and (4) a severe neurological behavior score.

### 2.6. Neurological Severity Score (NSS)

The neurological deficits were evaluated and recorded based on the following four parameters with minor modifications [28].

i. Zea-longa criteria

A 5-point scale was adopted as follows: 0, no neurological symptoms; 1, unable to fully stretch the contralateral forepaw; 2, tilting to the contralateral side while walking; 3, falling or limping on the contralateral side; 4, unable to walk spontaneously; and 5, death [29].

ii. Forelimb score

The mice were held by their tail; they were observed in the air and given a score as follows: 0, normal activity; 1, occasional flexion of the asymmetric forelimb; 2, asymmetric forelimb flexion; 3, flexion of the asymmetric forelimb and torso; and 4, no motor reaction [30].

iii. Circling score

Circling behavior was determined by observing the mice with only their tail and their forelegs in contact with the floor; the scores were: 0, no observable deficits; 1, flexion of the contralateral torso; 2, circling clockwise; 3, continuously spinning clockwise; and 4, no movement or reactions [30].

iv. Prehensile traction 4-grade score

A 0.5 cm diameter wire was fixed horizontally at a height 70 cm above the floor. After placing the mice forepaws onto the wire, the time until falling was measured: 0, hanging on the wire for over 5 s with its hind legs; 1, hanging on the wire for over 5 s; 2, hanging on the wire for 3~4 s; and 3, hanging on the wire for 0~2 s [31].

### 2.7. Primary Neuron Culture

Primary neuron cultures were prepared from the cortex of embryonic day 15 (E15) C57BL/6 mice. For the protocol, the brain was harvested, and the meninges and hippocampus were removed from the hemisphere under a microscope. The cortical neurons were collected in modified Eagle’s medium (MEM, Gibco/Thermo Fisher) with 1% glutamate and were washed once. The cortices were then treated with 0.125% trypsin at 37 °C for 10 min. The cell suspensions were filtered through a 40 μm cell strainer and plated on plate wells precoated with poly-D-lysine (P4707; Sigma-Aldrich). The collected cortical cells were counted and seeded at a density of 1.25 × 10^6^ neurons/well for in a 6-well plate and 3 × 10^5^ neurons/well for in a 24-well plate in Neurobasal medium containing 2% B27 supplement, 1% GlutaMAX (100×), and 1% antibiotic–antimycotic solution (all from Gibco/Thermo Fisher). One day after seeding the cells, the medium was replaced, and culture medium was then partially changed once every 3–4 days with freshly supplemented Neurobasal medium. The cultures were maintained at 37 °C in a humidified incubator of atmosphere mix containing 5% CO_2_.

### 2.8. Oxygen–Glucose Deprivation (OGD) Treatments

Oxygen–glucose deprivation (OGD) experiments were performed using a hypoxic humidified incubator that was flushed with a gas mixture of 90% N_2_, 5% H_2_, and 5% CO_2_ at 37 °C and contained an anaerobic gas mixture (also 90% N_2_, 5% H_2_, and 5% CO_2_), as described previously with minor modifications [32]. The primary neuronal cells were treated with vehicle (0.1% DMSO), PCE (10^−7^−10^−5^ g/mL), and PCCs (10^−6^ M) for 24 h prior to OGD insult. In the case of N2a cells, the cells were treated with vehicle (0.1% DMSO) and PCE (10^−7^−10^−5^ g/mL) for 24 h prior to OGD insult. To initiate OGD in neuronal cells, culture medium was replaced with glucose-free DMEM for the indicated time periods. After OGD treatment, the culture medium was replaced with maintenance medium, and the cells were returned to a humidified atmosphere incubator containing 5% CO_2_ at 37 °C for the reoxygenation period. Normoxic cells were incubated under normoxic-oxygenated DMEM containing glucose for the corresponding durations.

### 2.9. Ngb Promoter Assay

Ngb transcriptional activity was assessed as described previously [26]. SKNSH cells were seeded in a 96-well plate at an initial density of 2.5 × 10^4^ cells per well and cultured in 10% FBS in DMEM. After 10 h, with the cell confluence at about 40–50%, the culture medium was replaced with the experimental medium (2% FBS in phenol red-free DMEM). After the cell confluency reached about 70–80%, the vehicle (0.1% DMSO) and various concentrations of PCE (10^−8^−10^−6^ g/mL) and its compounds (10^−8^–10^−6^ M) were dissolved in experimental medium for 24 h. The cells were lysed after incubation for 15 min using 1× lysis buffer with shaking. Luciferase activities were measured using a luciferase reporter assay system (Promega, Madison, WI, USA). Luminescence was detected with a GloMax Explorer Multimode Detection System (Promega).

### 2.10. RNA Extraction and Quantitative Polymerase Chain Reaction (qPCR)

SKNSH cells were seeded in a 6-well plate and cultured in 10% FBS in DMEM. After 24 h, the vehicle (0.1% DMSO), PCE (10^−7^–10^−5^ g/mL), and PCCs (10^−6^ M) were dissolved in experimental medium for 24 h. Total RNA from SKNSH cells was extracted with Trizol reagent (Invitrogen Life Technologies, Carlsbad, CA, USA) or with the RNeasy mini kit (Qiagen, Hilden, Germany) according to the manufacturer’s instructions. cDNA was synthesized from 0.5 μg of total mRNA using ReverTra Ace qPCR RT Master Mix with gDNA Remover (FSQ-301, Toyobo, Osaka, Japan). The qPCR reactions were made up of the source cDNA, the primer pairs for each target gene, and the PCR SYBR Green kit reagent (Bio-Rad, Hercules, CA, USA). We examined the expression of the human Ngb gene. The quantitative gene expression reaction for the target gene was in a final volume of 20 μL and run in the QuantStudio 3 Real-Time PCR (Thermo Fisher) and the Applied Biosystems 7500 Real-Time PCR system (Thermo Fisher). The gene expression curves were analyzed on a log scale, and the threshold cycles were calculated mathematically. The quantitative target gene expression was calculated using the 2^−ΔΔCT^ method and normalized for experimental errors from several steps with the levels of the housekeeping gene, glyceraldehyde-3-phosphate dehydrogenase (GAPDH). All of the reactions were performed in triplicate. The chosen primers were (5′→3′): (Qiagen hNgb QuantiTect Primer Assay) hNgb forward, TGGAAGACCTGTCCTTCACTG; hNgb reverse, GAGCAGAGACTCACCCACTG; hGAPDH forward, GGCTGAGAACGGGAAGCTTGTCAT; hGAPDH reverse, CAGCCTTCTCCATGGTGGTGAAGA. The reaction for hNgb was performed at 95 °C for 10 min, followed by 40 cycles of 95 °C for 30 s, 60 °C for 30 s, and 72 °C for 30 s. 

### 2.11. Western Blot Analysis

In this experiment, primary neuronal cells were treated with the vehicle (0.1% DMSO), PCE (10^−7^−10^−5^ g/mL), and PCCs (10^−6^ M) for 24 h, and the animals were intraperitoneally administered the vehicle and PCE (250 mg/kg) for 3 consecutive days before ischemic stroke. Prior to protein extraction from brain tissue, coronal section samples stained with 2,3,5-triphenyltetrazolium chloride (TTC) were used on day 1 post-stroke. This provided a distinction between the two parts, the core and penumbra, in the infarct area. The animal experiment was conducted by distinguishing the core and penumbra area. Whole-cell proteins were extracted from the primary neuron cells and brain tissue using lysis buffer (Cell Signaling, Danvers, MA, USA) supplemented with proteases and phosphatase inhibitors (Sigma-Aldrich). After homogenizing and clearing the lysates, the obtained protein lysates were quantified using Pierce BCA Protein Assay Kit (Thermo Fisher). Equal amounts of proteins were separated via sodium dodecyl sulfate–polyacrylamide gel electrophoresis (SDS-PAGE) in 12% gels at 80 V for 2 h and transferred onto PVDF membranes (Millipore, Burlington, MA, USA) for 1.5 h at 100 V. The PVDF membrane was then blocked in 5% bovine serum albumin (BSA) in phosphate-buffered saline (PBS) buffer containing 0.1% Tween-20 (T-PBS) at room temperature (RT) for 1 h. Subsequently, the membrane was incubated at 4 °C with the primary antibodies overnight. Next, the membrane was washed with T-PBS, followed by incubation with horseradish peroxidase (HRP)-conjugated secondary antibodies at RT for 1 h. The positive signals were then visualized by the enhanced chemiluminescent kit (Dharmacon/Thermo Scientific, Rockford, IL, USA) using the Amersham Imager 600 apparatus (GE Healthcare, Chicago, IL, USA). The protein band intensities were normalized against β-actin (Sigma-Aldrich) as the loading control. The list of primary antibodies and their dilutions are as follows: Ngb (1:1000, RD181043050, BioVendor, Asheville, NC, USA) (1:1000, 13499-1-AP, Proteintech., Manchester, UK), p38 (1:1000, cs9212, Cell Signaling), phospho-p38 (p-p38, 1:1000, cs9216, Cell Signaling), phospho-AKT (p-AKT, 1:1000, cs9271, Cell Signaling), caspase 3 (1:1000, cs9662, Cell Signaling), cleaved caspase-3 (cCasp3, 1:1000, cs9661, Cell Signaling), Bcl-2 (1:1000, 610539, BD Biosciences, USA), and phospho-Bad (1:1000, cs9295, Cell Signaling).

### 2.12. Cytotoxicity Assay

Cell death was evaluated using the lactate dehydrogenase (LDH) assay kit (BioVision, Milpitas, CA, USA). After OGD and reoxygenation, the culture medium was transferred to a 96-well plate in duplicate and incubated with LDH catalyst and dye mix at 37 °C in the dark. After 30 min, the absorbance was measured for each sample at 490 nm with the reference wavelength at 600 nm using a GloMax Explorer Multimode Detection System (Promega).

### 2.13. ROS Measurements

Intracellular ROS production was detected using the fluorescence probe 2′,7′-dichlorodihydrofluorescein diacetate (H2DCFDA, D399, Thermo Fisher), which is oxidized to the highly fluorescent 2′,7′-dichlorofluorescein (DCF) when intracellular ROS are generated. H2DCFDA was dissolved in DMSO to obtain a 10 mM stock solution. After treatment with different agents, neuronal cells and brain sections were washed and loaded with 10 μM H2DCFDA at 37 °C for 30 min in the dark. The cells were washed and fixed with 4% paraformaldehyde (PFA) (Biosesang, Seongnam, Korea). The brain sections were also washed twice. The cells and sections were counterstained with 4′,6-diamidino-2-phenylindole (DAPI) (Vectashield mounting medium with DAPI, H-1500, Vector Laboratories, Burlingame, CA, USA). ROS production was detected with a confocal laser scanning microscope (LSM-800, Zeiss, Munich, Germany) at excitation/emission wavelengths of 488/525 (for DCF) and 358/461 nm (for DAPI). In the collected images, fluorescence intensities indicating DCF-positive cells were quantified using the ImageJ software (NIH, Bethesda, MD, USA).

### 2.14. Determination of Antioxidant Enzyme Activity

N2a cells were cultured in a 6-well plate at a density of 2 × 10^5^ cells per well for 24 h. The cultured cells were treated with the vehicle (0.1% DMSO) and PCE (10^−7^–10^−5^ g/mL) for 24 h. After 24 h, OGD insult was performed for 4 h and followed by reoxygenation for 24 h. The activities of the two antioxidant enzymes (glutathione (GSH) and catalase (CAT)) in N2a cells were assessed using commercial assay kits (Biomax, Seoul, Korea). Briefly, for the GSH assay, the cells were washed twice and scraped from the plate, and then a density of 1–5 × 10^6^ cells were collected and washed. After resuspension with ice-cold 5% metaphosphoric acid (MPA), the cells were homogenized and followed by centrifugation at 12,000 rpm and 4 °C for 10 min. For the CAT assay, cells at a density of 1–2 × 10^6^ cells/mL were collected and washed. The cells were homogenized, followed by centrifugation to remove the debris. In both the GSH and CAT assays, after the supernatant was collected, antioxidant enzyme activities were determined according to the manufacturer’s instructions. The samples for the GSH assay were measured by the kinetic method with absorbance at 412 nm, and fluorescence for the CAT assay was measured using a GloMax Explorer Multimode Detection System (Promega) at excitation/emission wavelengths of 520/590 nm.

### 2.15. Estimations of Infarct Volume by Cresyl Violet and 2,3,5-Triphenyltetrazolium Chloride (TTC) Staining

All experimenters performing the assessments were blinded to their experimental groups. The mice were cardioperfused with saline, followed by fixation using 4% PFA. Their brains were removed and frozen for 1 h at −80 °C. The obtained brains were cryosectioned coronally into 30 μm thick slices. Infarct volume was evaluated using a protocol described previously with minor modifications [33]. Briefly, 13 serial coronal sections were made; these were 30 μm thick and taken at 600 μm intervals from the frontal pole. The sliced sections were rehydrated and stained with 0.1% cresyl violet (Sigma-Aldrich) in distilled water and then dehydrated. The infarct areas were measured using the ImageJ software (NIH). The infarct volumes were calculated by taking the sum of infarct areas from all of the sections and multiplying by 600 µm. For TTC staining, anesthetized animals were decapitated, and their brains were carefully removed and sliced coronally into six 1 mm thick slices using a mouse brain matrix guide. The coronal slices were then incubated in 2% TTC solution (Sigma-Aldrich) at 37 °C for 10 min [34]. The infarct volumes were measured using the ImageJ software (NIH). The total infarct volume was obtained as the sum of the infarctions of all slices multiplied by the thickness of the brain sections. The TTC-stained brain regions were dissected and immediately frozen at −80 °C.

### 2.16. Immunohistochemistry

Immunohistochemistry was performed on 30 µm coronal sections. The brain sections were washed twice for 10 min and blocked with 5% normal horse serum for 1 h. The sections were incubated with primary antibodies against Ngb (Proteintech), phospho-p38 (Cell Signaling), cleaved caspase-3 (Cell Signaling), manganese superoxide dismutase (mnSOD (SOD2), SOD110, StressGen, Victoria, BC, Canada), and gp91phox (BD611414, BD Biosciences). Staining was also performed with antineuronal nuclei (NeuN) directly conjugated to Alexa Fluor 488 (MAB377X, Millipore). The primary antibody incubations were performed at 4 °C overnight. After incubation of primary antibodies, the sections were washed, and for the unlabeled antibodies, the samples were then incubated with Alexa Fluor conjugated secondary antibodies (Invitrogen/Thermo Fisher) for 1 h at 25 °C. The sections were counterstained with DAPI, and the fluorescence signals were examined using a confocal laser scanning microscope (LSM-800, Zeiss) at excitation/emission wavelengths of 495/519 (for Alexa Fluor 488), 590/617 (for Alexa Fluor 594), and 358/461 (for DAPI) nm.

### 2.17. Statistical Analysis

The data are expressed as mean ± standard error of the mean (SEM). Statistical analysis was performed using GraphPad Prism software (GraphPad, San Diego, CA, USA). Student’s *t*-tests were used to compare the mean values of two independent groups for one variable. Comparisons between multiple groups were conducted by one-way analysis of variance (ANOVA) followed by the Holm–Sidak method and Tukey’s multiple comparison tests. Differences with *p*-values of *p* < 0.001, *p* < 0.01, and *p* < 0.05 were considered significant.

## 3. Results

### 3.1. PCE and Its Effective Compounds Increase Ngb Expression in Neuronal Cells

**Figure 1:**
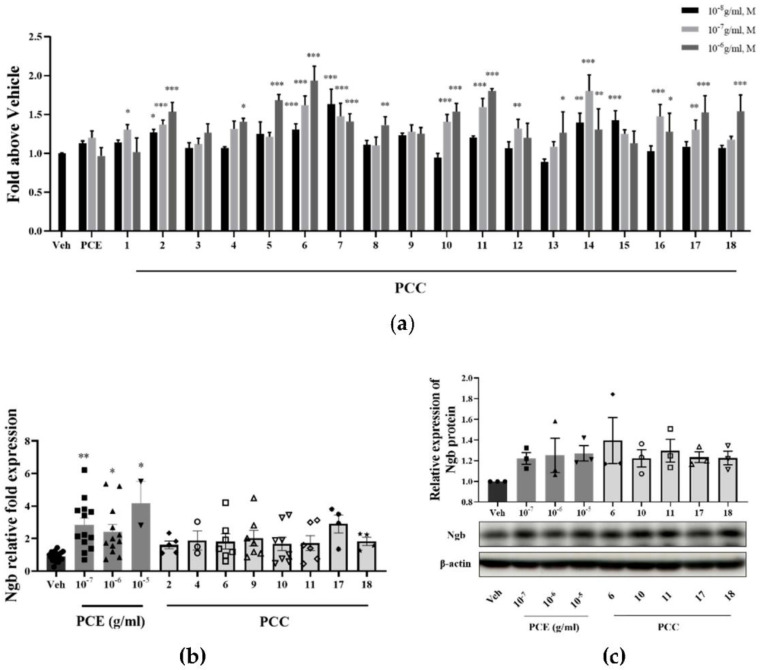
PCE and PCCs induce Ngb transcriptional activity and increase Ngb protein levels in treated neuronal cells. (**a**) Increased Ngb promoter activity levels from PCE and PCCs by luciferase reporter assay in SKNSH cells. SKNSH cells were treated with various concentrations of PCE (10^−8^−10^−6^ g/mL, *n* = 10) and PCCs (10^−8^−10^−6^ M, *n* = 10) for 1 day. Results are expressed as the relative luciferase value (Veh, *n* = 20). (**b**) Levels of Ngb mRNA were increased by PCE (10^−7^−10^−5^ g/mL) and PCCs (10^−6^ M) in SKNSH cells. The ratio of target gene expression to that of GAPDH housekeeping gene was determined by qPCR as a quantitative measure of gene expression. Ngb mRNA levels were significantly increased by 10^−5^ g/mL PCE and leucoside (Veh, *n* = 18; PCE, *n* = 13; PCCs, *n* = 8). (**c**) Protein levels of Ngb were induced by PCE (10^−7^−10^−5^ g/mL) and selected PCCs (10^−6^ M) in primary neuronal cells (*n* = 4). The mean values in triplicate experiments are presented as SEM. *** *p* < 0.001, ** *p* < 0.01, * *p* < 0.05 compared with the vehicle control, determined by the Holm–Sidak method following a one-way analysis of variance.

Transcriptional activity, mRNA, and protein levels of Ngb were analyzed for Ngb expression changes due to PCE and its compounds (PCCs). To screen for an effect on Ngb transcription levels, a luciferase reporter assay for Ngb was performed in SKNSH cells (Figure 1a). PCE induced Ngb transcriptional activity at a concentration of 10^−7^ g/mL. Among the particular PCE components, genistin, prunetinoside, prunetin, naringenin, sakuranetin, prunin, quercetin 3-sambubioside, hyperoside, leucoside, and gentisic acid 5-O-β-D-(6′-O-trans-4-coumaroyl)-glucopyranoside, a novel compound, significantly increased Ngb transcriptional activity by up to 2-fold at a concentration of 10^−6^ M (Figure 1a). In general, most of the component compounds increased Ngb transcription and had dose-dependent responses. These results indicate that PCE and most of its component compounds, including prunetinoside, prunetin, sakuranetin, and prunin, had dose-dependent Ngb transcriptional induction activity.

Based on the results of the Ngb promoter assay, the PCE and eight compounds were selected for evaluating Ngb mRNA level changes in SKNSH cells (Figure 1b). All treatment concentrations of PCE significantly increased Ngb mRNA levels by more than two-fold in SKNSH cells. The selected component compounds also increased Ngb mRNA levels in these cells. Of note, leucoside increased the levels of Ngb mRNA by more than two-fold, a level higher than that seen for treatment with PCE. From the transcriptional activity changes for Ngb, among the PCCs, five compounds were selected for assaying protein level changes in Ngb in the test cells. Increased protein Ngb levels in the treated primary cultured neuronal cells were observed for these compounds (Figure 1c), as treatment with concentrations of 10^−7^ to 10^−5^ g/mL of PCE and each PCC significantly increased Ngb protein expression by as much as 1.2-fold in those cells. Overall, these results indicate that PCE and its active compounds, including a newly described compound, increases Ngb transcriptional activity as well as total Ngb protein levels in neuronal cells.

### 3.2. PCE and Its Component Compounds Alleviate OGD/Reoxygenation (OGD/R)-Induced Injury in Primary Neurons

**Figure 2:**
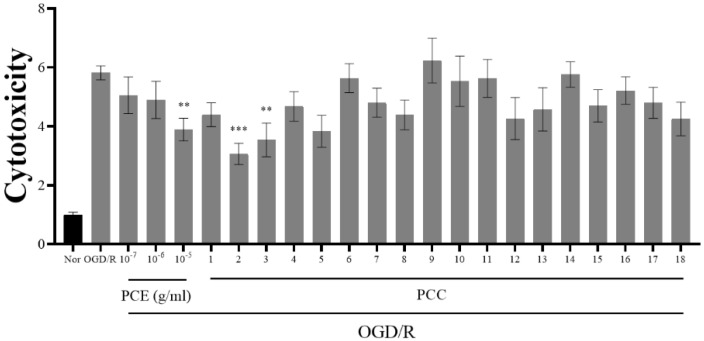
Reduced cytotoxicity due to PCE and PCCs from an assay of LDH release in primary neuron cells. Primary cultured mouse neuron cells were pretreated with various concentrations of PCE (10^−7^–10^−5^ g/mL) and PCCs (10^−6^ M) for 24 h and then exposed to 2 h of OGD, followed by 24 h of reoxygenation. Cell cytotoxicity was assessed by the LDH assay (readings at 490 and 600 nm). The mean values in triplicate experiments are presented as SEM. *** *p* < 0.001, ** *p* < 0.01, compared with the Nor, determined by Tukey’s multiple comparison test following a one-way analysis of variance (Nor and OGD/R, *n* = 100; PCE and PCCs, *n* = 20).

It has been reported that Ngb prevents neuronal cell death due to OGD/R [35]. Our results indicate that PCE and PCCs upregulate Ngb levels; therefore, we analyzed whether PCE and PCCs could alleviate OGD/R-induced cytotoxicity in primary neuronal cells (Figure 2). The primary cultured neurons were treated with PCE and PCCs for 24 h prior to 2 h OGD insult with reperfusion for 24 h after OGD. In the OGD/R group, the measured cytotoxicity was increased by about six-fold compared with the normoxia control (Nor) cells. Pretreatment of the cells with PCE at the 10^−5^ g/mL dose significantly reduced cell death by more than 30%. Similarly, the PCC compounds sakuranin and gentisic acid 5-O-β-D-(6′-O-trans-4-coumaroyl)-glucopyranoside also decreased neurotoxicity by approximately 30% in OGD/R-exposed neurons. Pretreatment with genistin and genistein 5-glucoside also significantly decreased neurotoxicity in OGD/R-exposed neurons (by approximately 47% and 39%, respectively). These results indicate that PCE and some of its component compounds can significantly ameliorate OGD/R-induced cell death in primary cultured neurons.

### 3.3. PCE Exhibits Antioxidant Activities in Neurons against Hypoxic Injury In Vitro

**Figure 3:**
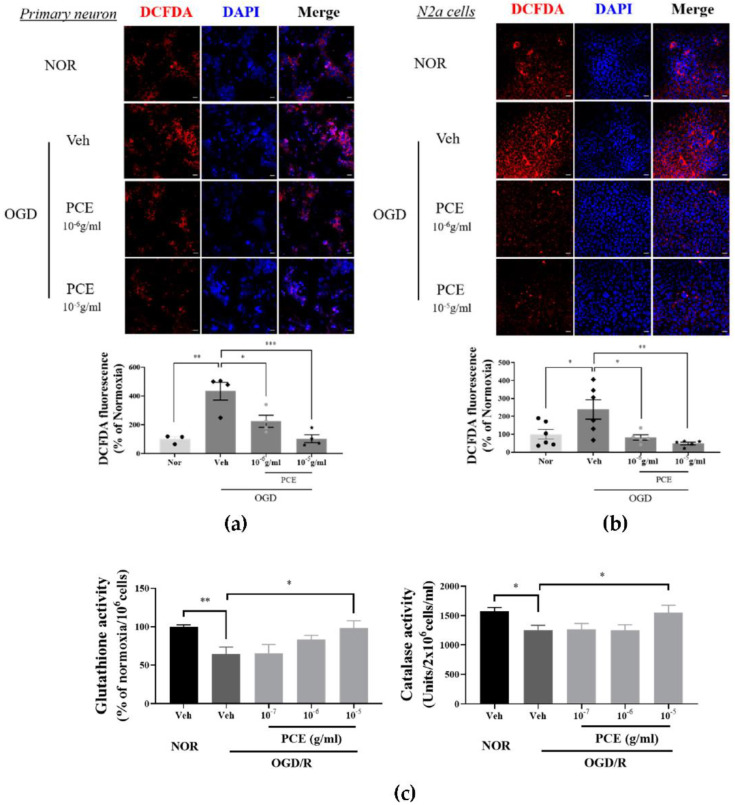
Antioxidant properties of PCE in OGD-induced neuronal cells. (**a**,**b**) Representative immunofluorescence images of DCFDA (red) staining showing superoxide levels in neuronal cells exposed to 2 h OGD/0.5 h R and 4 h OGD/0.5 h R in primary cortical neuronal cells and N2a cells, respectively. Primary cortical neuronal cells were treated with two concentrations of PCE (10^−6^ and 10^−5^ g/mL) before OGD/R. PCE inhibited ROS production in OGD/R-exposed neuronal cells. (**c**) Estimation of GSH and CAT enzyme activities in N2a cells exposed to 4 h OGD/24 h R. The antioxidant enzyme activities were decreased after OGD/R insult in N2a cells, whereas PCE increased its GSH and CAT activities as compared to the vehicle treatment in OGD/R-exposed N2a cells. The mean values in triplicate experiments are presented as SEM. White scale bar = 20 μm. *** *p* < 0.001, ** *p* < 0.01, * *p* < 0.05, determined by Student’s *t*-test for homogeneous variance and one-way ANOVA, followed by Tukey’s multiple comparison test (N2a, *n* = 7, primary neuron, *n* = 4).

It has been reported that Ngb acts as an ROS scavenger in oxidative stress situations [36]. A possible role for PCE, being an Ngb activator for antioxidant activity, was investigated for increased intracellular ROS levels induced by OGD/R. DCFDA, a fluorescence probe, was used for ROS detection. In primary cultured neurons, OGD/R increased ROS levels by more than 4-fold compared with Nor controls, whereas intracellular ROS production in PCE-treated neurons was decreased by 48.2% and 76.1% at 10^−6^ g/mL and 10^−5^ g/mL levels, respectively, in a dose-dependent manner (Figure 3a). Similar results were observed in N2a cells. PCE-treated N2a cells showed a reduction in the levels of ROS production by 69.9% and 82.8% at concentrations of 10^−6^ to 10^−5^ g/mL, respectively, matching similar trends in ROS reduction by PCE in primary cultured neurons (Figure 3b). To evaluate the antioxidant properties of PCE against oxidative stress in vitro, intracellular GSH and CAT activities were measured in OGD/R-exposed N2a cells (Figure 3c). As shown in Figure 3c, OGD/R-induced hypoxic damage caused a significant decrease in GSH and CAT activities compared with those in the normoxia control group. GSH activity was significantly increased by 1.5-fold in the PCE treatment (10^−5^ g/mL) compared with that in the vehicle treatment in OGD/R-exposed N2a cells, which increased in a dose-dependent manner. CAT activity was also significantly increased by 1.2-fold in the PCE treatment (10^−5^ g/mL) compared with that in the vehicle treatment in OGD/R-exposed N2a cells. In particular, PCE at a concentration of 10^−5^ g/mL showed increased activities of GSH and CAT, suggesting that PCE has antioxidant properties against hypoxia-induced oxidative stress. These findings indicate that decreased cytotoxicity by PCE is closely related to reduced ROS levels and increased antioxidant enzymes in neurons after oxidative stress, with PCE mediating the effects in multiple cell types.

### 3.4. PCE Promotes Antioxidant Activity in the Brain after tMCAO in Mice

**Figure 4:**
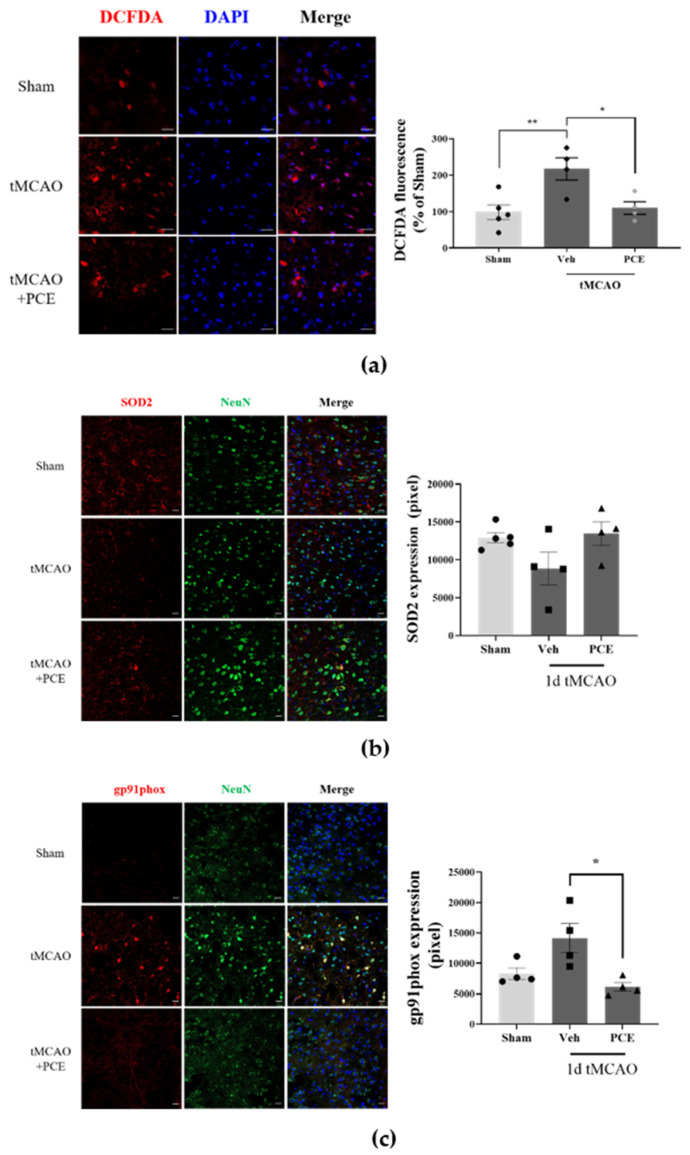
Antioxidant effects of PCE in the ischemic brain. Representative images of mouse brain stained with DCFDA, SOD2, and gp91phox. (**a**) DCFDA fluorescence was assessed along with the LDH assay following 2 h OGD and 0.5 h reoxygenation. PCE significantly decreased OGD-induced neurotoxicity. (**b**) The expression of SOD2 was decreased in the ischemic area after tMCAO, while PCE rescued its expression compared with the vehicle-treated tMCAO. (**c**) The expression of gp91phox was increased by ischemic stroke, whereas its expression in the PCE-treated tMCAO group was decreased compared with that in the vehicle-treated tMCAO group. The data represent mean ± SEM. White scale bar = 20 μm. ** *p* < 0.01, * *p* < 0.05, analyzed using one-way ANOVA followed by the Tukey’s multiple comparison test (DCFDA, SOD2, *n* = 5; gp91phox, *n* = 4).

We found that neuronal cells treated with PCE exhibited decreased cytotoxicity and rescued activities of antioxidant enzymes in vitro. To confirm this antioxidant effect of the PCE in vivo, first, we determined the intracellular ROS production in the ischemic brain area of tMCAO-treated mice. At 24 h post-tMCAO, ROS production around damaged cells accumulated to more than 2-fold compared with the sham-operated group in the same brain area (Figure 4a). By contrast, the PCE-treated tMCAO group had significant reductions in accumulated ROS, the levels of which were half of those in the vehicle-treated tMCAO group in both the core and penumbra areas. In fact, ROS production in the PCE-treated group was similar to that of the sham-operated group. In order to determine the expression of antioxidant enzymes induced by PCE, we also measured the effects of PCE on SOD2 levels in ischemic brains in the tMCAO model, which showed the antioxidant properties of PCE via SOD2 expression in the ischemic area. The expression of SOD2 was rescued by PCE administration, and this level was similar to that of the sham control group (Figure 4b). In addition, we found that PCE ameliorated the active superoxide production system through the reduction in gp91phox, a plasma membrane subunit of nicotinamide adenine dinucleotide phosphate (NADPH) oxidase (Nox) (Figure 4c). The PCE-treated tMCAO group had half of the increased expression of gp91phox in the ischemic area compared with the vehicle-treated tMCAO group. These results indicate that treatment with PCE provides an antioxidant effect by reducing intracellular ROS levels produced after hypoxic injury and by elevating antioxidant enzymes in both neuronal cells and brain tissue. These findings suggest that PCE upregulates Ngb and that the regulation of antioxidant enzymes by PCE might be closely associated with PCE-related antioxidant activity.

### 3.5. PCE Improves Neurological Deficits and Infarct Volume following tMCAO in Mice

**Figure 5:**
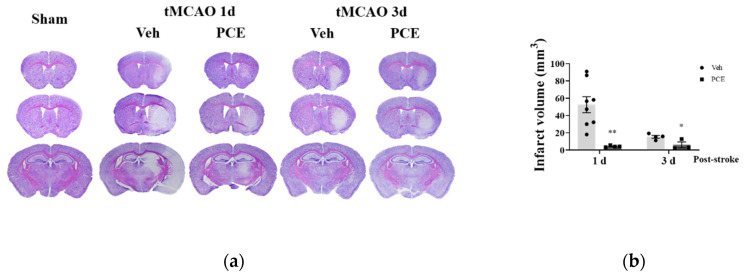
Pretreatment PCE group has reduced infarct volume in ischemic stroke mouse brain. (**a**) Representative cresyl violet-stained sections of mouse brain on days 1 and 3 post-stroke. (**b**) Graph displays the quantification of measured infarct volumes. PCE treatment reduced the infarct volume by approximately 90.6% and 60.1% compared with the vehicle group after 1 and 3 days post-tMCAO, respectively (1d, *n* = 8; 3d, *n* = 4). Values are mean ± SEM. ** *p* < 0.01, * *p* < 0.05, compared with the vehicle-treated tMCAO group, determined by Student’s *t*-test.

**Figure 6:**
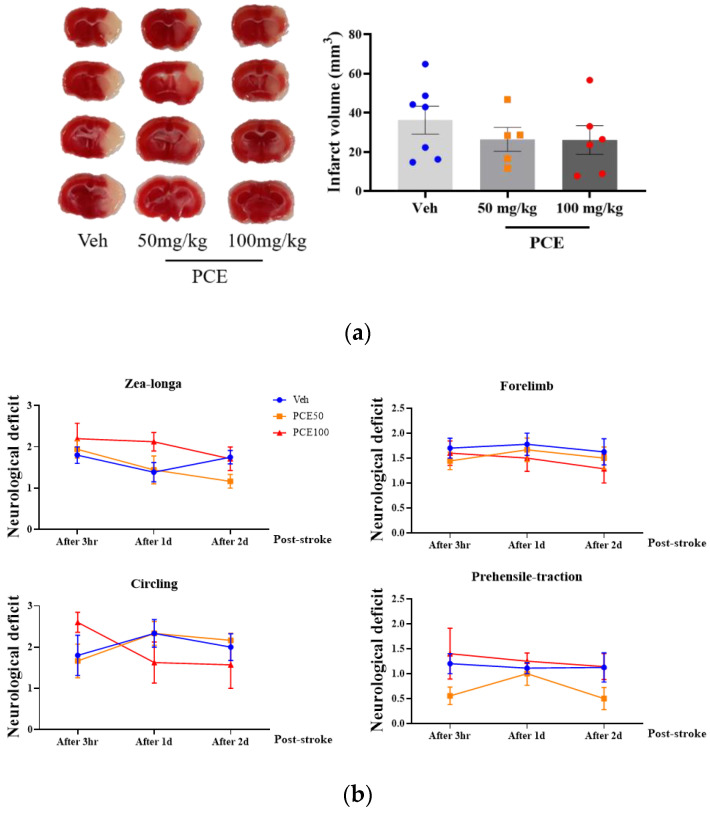
Post-treatment PCE diminishes infarct volume in ischemic stroke mouse model brain. (**a**) Representative 2,3,5-triphenyltetrazolium chloride (TTC)-stained sections of mouse brain 2 days post-stroke. Graph shows quantification of infarct volume. (**b**) Plots of four neurological subset test scores 3 h after perfusion. Post-treatment with PCE reduced the infarct volume by approximately 27% compared with the vehicle group and improved the neurological outcomes (Veh, *n* = 7; 50 mg/kg, *n* = 5; 100 mg/kg, *n* = 6). Values are mean ± SEM.

To determine the functional extent of PCE-mediated protective effects against ischemia/reperfusion (I/R) injury in mice, on days 1 and 3 after the induction of ischemic stroke, we measured the infarct area size by Nissl staining (Figure 5a). Pretreatment of the mice with PCE (250 mg/kg, i.p) for 3 consecutive days reduced the infarct volume by approximately 90.6% and 60.1%, respectively, compared to the vehicle group on days 1 and 3 post-tMCAO (Figure 5b). We investigated four different types of neurological deficits and infarct volume changes in the tMCAO mouse model (Table 2). The neurological impairments increased after 1 day post-stroke, while these neurological deficits were improved by PCE pretreatment of the mice. The forelimb score of the PCE-pretreated tMCAO group was 1, the circling score was 0.3 ± 0.21, and the prehensile traction score was 0.3 ± 0.18. These scores in the PCE-pretreated tMCAO group were significantly lower than those in the vehicle-treated tMCAO group. For the post-treatment of mice with PCE, they were intraperitoneally administered once after stroke, and the effects were investigated 2 days later (Figure 6a,b). Post-treatment with PCE (50 mg/kg) led to a reduction in the cerebral infarct volume and improved neurological scores compared to the vehicle-treated tMCAO group (Figure 6a). These results indicate that administration of PCE improved neurological outcomes and attenuated cerebral infarct size in both pre- and post-treated animals in the acute cerebral ischemia mouse model.

### 3.6. PCE Prevents Cerebral Ischemic Damage by Increasing Ngb Expression and Decreasing Activated p38 in the Penumbra Area

**Figure 7:**
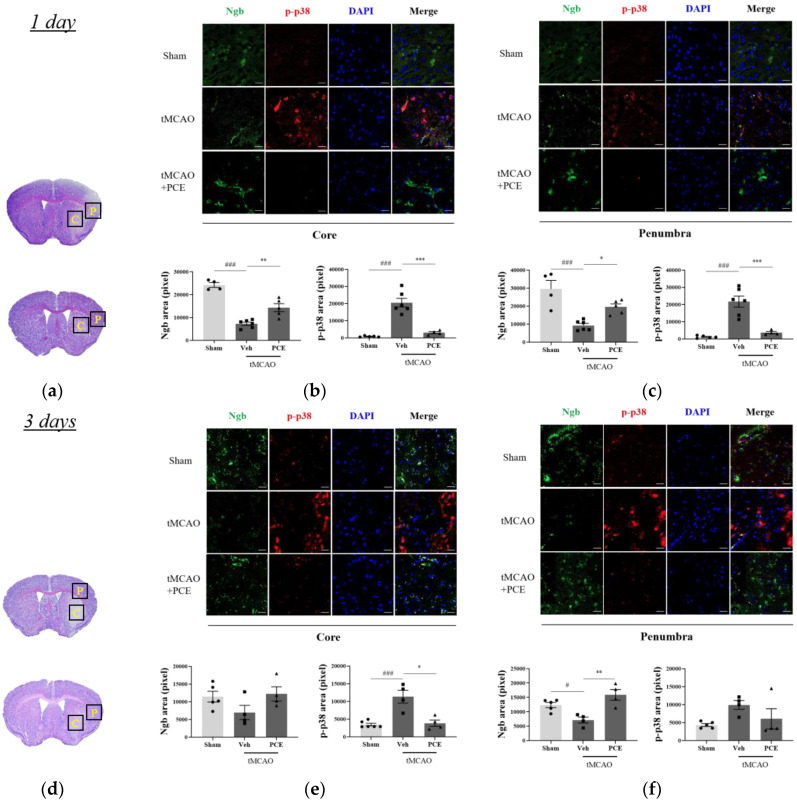
Immunofluorescence staining indicates changes in Ngb and activated p38 levels in the ischemic stroke brain. (**a**,**d**) Representative images of Nissl staining of the areas that were selected for quantifying Ngb and p-p38 in the ipsilateral brain area 1 day (**a**–**c**) and 3 days (**d**–**f**) post-stroke, respectively. Black solid line and yellow-labeled boxes (1 × 1 mm^2^ square field) outline the core and penumbra (C and P) infarct regions. (**b**,**c**,**e**,**f**) Representative images and quantification of Ngb (green) and p-p38 (red) staining in the ipsilateral area post-tMCAO in mice. Treatment with PCE resulted in significant increases in Ngb and decreases in activated p38 in comparison with the vehicle-treated tMCAO group. Data are presented as mean ± SEM. White scale bar = 20 μm. ***, ### *p* < 0.001, ** *p* < 0.01, *, # *p* < 0.05. Statistical significance was determined using one-way ANOVA, followed by Tukey’s multiple comparisons test (1 day post-stroke, *n* = 5; 3 days post-stroke, *n* = 4).

In order to detail the neuroprotective effects of PCE as an activator of Ngb, we measured the signaling pathway changes related to oxidative stress after cerebral ischemia. It has been reported that p38 mitogen-activated protein kinase (MAPK) is activated by oxidative stress and that it is linked to the activation of caspase-3-mediated apoptosis [37]. We evaluated the effect of PCE on Ngb expression levels as well as activation of p38 in the core and penumbra areas on days 1 and 3 post-stroke (Figure 7). Prior to immunostaining of the brain samples, the infarct area was identified in the core and penumbra areas by Nissl staining of the brain sections (Figure 7a,d). The results indicate that the tMCAO group had decreased levels of Ngb expression in the core area compared to the sham-operated group (Figure 7b,c). However, PCE treatment of the mice significantly increased Ngb expression by about 2-fold compared with the vehicle-treated tMCAO group, particularly in the penumbra area. In addition, the activated p38 MAPK levels were dramatically increased in the core area in response to cerebral ischemia compared to those in the sham-treated group, while the PCE-treated tMCAO group had markedly reduced levels of activated p38; these levels were decreased by about 80% and 40% compared to the vehicle-treated tMCAO group on days 1 and 3 post-stroke, respectively (Figure 7b–f). These results also support the negative correlation of Ngb levels and activated p38 after stroke in the core area on days 1 and 3 post-stroke (Figure 7b,e). 

**Figure 8:**
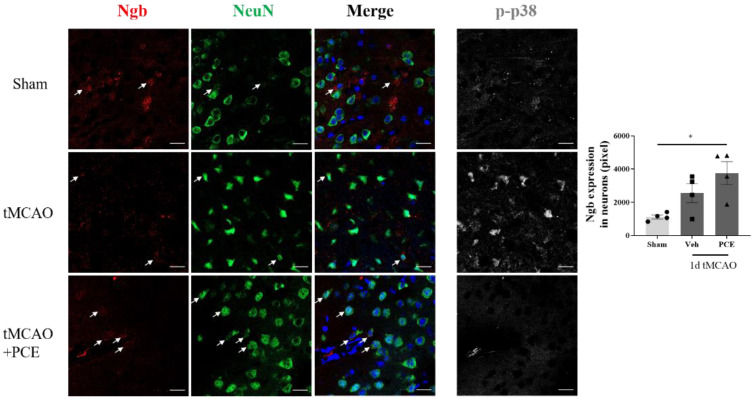
PCE treatment increases Ngb levels in neurons in the ischemic area. Representative immunofluorescence images and quantification of colocalization with Ngb (red) and NeuN (green) staining in the ipsilateral area. Immunofluorescence images of activated p38 (white) are also shown as a reference. The numbers of Ngb-expressing neurons decreased after ischemic stroke. PCE treatment exhibited increased numbers of Ngb-expressing neurons. Colocalization of Ngb and neurons is indicated by white arrows. Data are presented as mean ± SEM. White scale bar = 20 μm. * *p* < 0.05. Statistical significance was determined using one-way ANOVA, followed by Tukey’s multiple comparison test (*n* = 4).

To estimate the PCE-induced changes in Ngb levels in neurons in the ischemic area, we co-stained the samples for NeuN, a neuronal marker (Figure 8). It was observed that damaged neurons in the ischemic area had strong p38 activation and also showed relatively low levels of Ngb. In contrast, PCE treatment of the mice reduced activated p38 and upregulated Ngb in neurons in the ischemic area.

**Figure 9:**
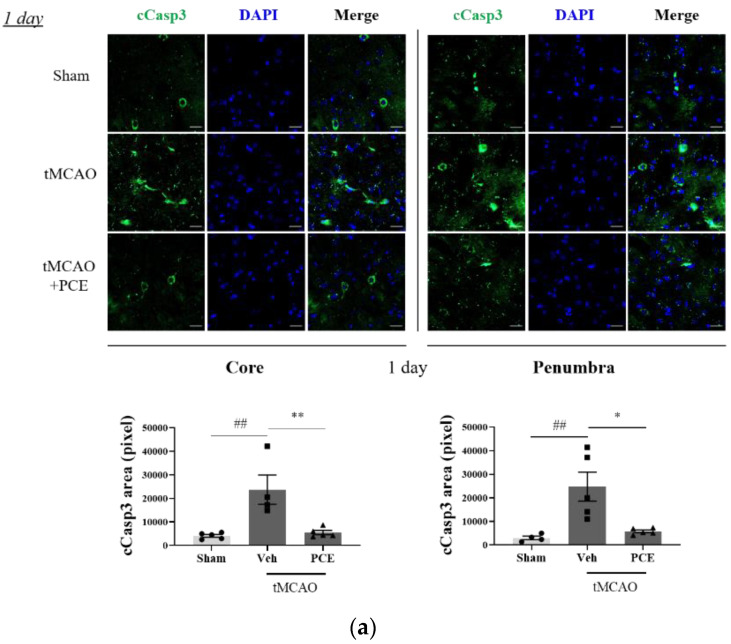

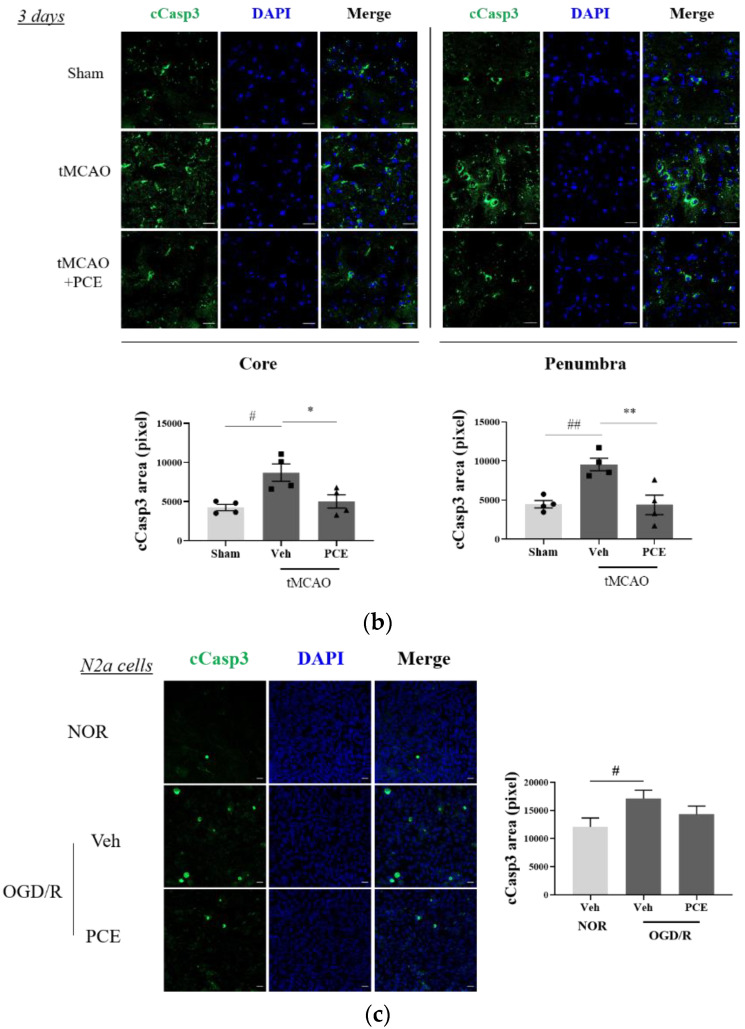
Immunofluorescence staining indicates changes in cleaved caspase-3 (cCasp3) levels in PCE-treated tMCAO samples. (**a**,**b**) Representative images and quantification of cCasp3 (green) staining in the core and penumbra 1 day (**a**) and 3 days (**b**) after stroke induction, respectively. After ischemic stroke, the vehicle-treated tMCAO group showed increased cCasp3 levels compared with the sham-operated group; cCasp3 levels were markedly decreased by the PCE treatment in both the core and penumbra areas. In particular, 3 days post-stroke, the levels of cCasp3 increased compared to those on day 1, and the significant decreases due to PCE treatment were retained until 3 days post-stroke. (**c**) The expression of cCasp3 in N2a cells exposed to OGD/R insult. cCasp3 expression increased in vehicle treatment, while PCE treatment (10^−5^ g/mL) decreased cCasp3 expression in N2a cells exposed to OGD/R insult. Data are presented as mean ± SEM. White scale bar = 20 μm. **, ## *p* < 0.01, *, # *p* < 0.05. Statistical significance was determined by Tukey’s multiple comparison test following one-way analysis of variance (Sham and tMCAO, *n* = 5; tMCAO-PCE, *n* = 4; N2a cells, *n* = 2).

To investigate whether the neuroprotective effect of PCE was associated with increased antiapoptotic activity, the expression of cleaved caspase-3 (cCasp3) was also examined as a signal for increased apoptosis due to oxidative stress. Caspase-3 is rapidly activated during acute cerebral ischemia, and it generally contributes to cell death in both the core and penumbra areas after ischemia induction [38]. In the tMCAO group, cCasp3 expression increased 5-fold and 2-fold on days 1 and 3 post-stroke, respectively, when compared to the sham-treated group. By comparison, the PCE-treated group had decreased cCasp3 expression in both their core and penumbra areas (Figure 9a). In this analysis, the expression levels of cCasp3 on day 3 post-stroke induction were still high in the vehicle-treated group, but significant reductions were retained by the PCE treatment until day 3 post-stroke (Figure 9b). In order to confirm the role of PCE in antiapoptotic activity in vitro, N2a cells were stained with cCasp3 after 4 h OGD and 24 h reoxygenation (Figure 9c). After OGD/R insult, cCasp3 expression significantly increased by about 1.5-fold compared with the normoxia group. However, increased cCasp3 expression was decreased by about 20% when OGD/R-exposed N2a cells were treated with PCE (10^−5^ g/mL). These findings suggest that the effects of PCE on increasing Ngb levels and attenuating activated p38 levels are also associated with the antiapoptotic benefits of PCE in response to oxidative stress.

### 3.7. PCE Promotes Survival-Associated Signals in tMCAO Brain

**Figure 10:**
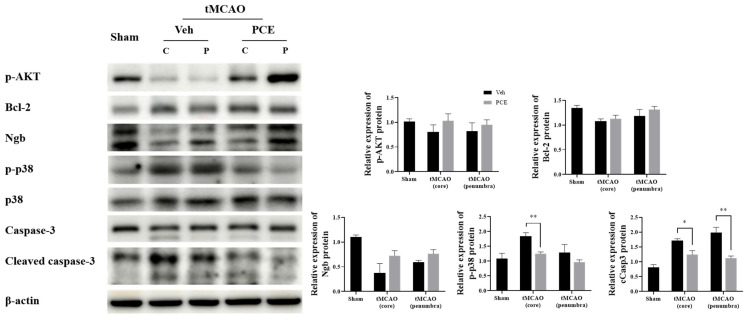
Changes are seen in the levels of prosurvival/apoptosis-associated molecules in the ischemic area due to PCE treatment. Expressions of antiapoptotic molecules p-AKT and Bcl-2 in the core region of ischemic stroke were higher in the PCE-treated tMCAO group than in the vehicle-treated tMCAO group. In the core and penumbra of the tMCAO vehicle-treated group, the expression of Ngb was decreased, and levels of activated p38 and cCasp3 were elevated; these effects were mostly reversed by PCE treatment. The mean values in triplicate experiments are presented along with SEM. Statistical significance was determined using the Holm–Sidak method (*n* = 4), ** *p* < 0.01, * *p* < 0.05.

Ischemic injury produces oxidative stress, which then suppresses the expression of survival genes and induces the expression of genes related to cell death [39]. Therefore, we examined the expression of certain molecules associated with oxidative stress in vivo and analyzed whether activation of oxidative-related signaling is obviated in the PCE group. The protein expression levels of prosurvival/antiapoptotic molecules, p-AKT and Bcl-2, in the core region of the post-stroke brain were then seen to be higher in the PCE-treated group than in the vehicle-treated group after tMCAO (Figure 10). Next, we examined changes in the protein expression level of Ngb and oxidative stress-related molecules in the ischemic core and penumbra areas. The expression levels of Ngb were decreased, and those of activated p38 and cleaved Casp3 were elevated in the vehicle-treated tMCAO group, with the changes blunted in the PCE-treated tMCAO group, particularly in the penumbra area. These results indicate that in the infarct areas, there are decreases in levels of survival-related molecules and Ngb expression, but PCE treatment of the mice upregulates both the prosurvival signals and Ngb levels in the core and penumbra areas.

In order to detail the neuroprotective effects of PCE as an activator of Ngb, we measured the signaling pathway changes related to oxidative stress after cerebral ischemia. It has been reported that p38 mitogen-activated protein kinase (MAPK) is activated by oxidative stress and that it is linked to the activation of caspase-3-mediated apoptosis [36]. We evaluated the effect of PCE on Ngb expression levels as well as the activation of p38 in the core and penumbra areas on days 1 and 3 post-stroke (Figure 7). Prior to immunostaining of the brain samples, the infarct area was identified in the core and penumbra areas by Nissl staining of the brain sections (Figure 7a,d). The results indicate that the tMCAO group had decreased levels of Ngb expression in the core area compared to the sham-operated group (Figure 7b,c). However, PCE treatment of the mice significantly increased Ngb expression by about 2-fold compared with the vehicle-treated tMCAO group, particularly in the penumbra area. In addition, the activated p38 MAPK levels were dramatically increased in the core area in response to cerebral ischemia compared to those in the sham-treated group, while the PCE-treated tMCAO group had markedly reduced levels of activated p38; these levels were decreased by about 80% and 40% compared to the vehicle-treated tMCAO group on days 1 and 3 post-stroke, respectively (Figure 7b–f). These results also support the negative correlation of Ngb levels and activated p38 in the core area after stroke on days 1 and 3 post-stroke (Figure 7b,e). 

## 4. Discussion

Despite the increasing incidence of ischemic stroke in the general population [40], the only FDA-approved and effective therapeutic approach post-stroke is to rescue the potentially recoverable infarct area through reperfusion using tissue plasminogen activator (tPA) [41]. In the current study, we propose PCE as a novel drug for treating ischemic stroke. The present findings demonstrate that the flavonoid-rich PCE upregulates levels of Ngb, an intracellular neuroprotective agent in oxidative stress, in OGD-exposed neurons and in ischemic stroke-induced mice. To the best of our knowledge, this is the first study to examine the effect of PCE on neuroprotection in a cerebral ischemia model.

We reported 18 compounds, including a novel compound, as constituents of PCE in a previous study [25]. A few studies have shown that flavonoid components upregulate Ngb promoter activity and its mRNA expression [26,42]. Therefore, we investigated whether PCE, as it contains abundant levels of flavonoids, would upregulate Ngb levels and act as a neuroprotectant from oxidative stress. Our results demonstrate that PCE and PCCs increased Ngb promoter activities as well as the mRNA levels and protein levels of Ngb. The upregulation of Ngb is thought to be brought about by the bioactive constituents of PCE, such as isoflavonoids and flavonoids. Among the 18 compounds referred to above in PCE, genistein, prunetinoside, prunetin, sakuranetin, prunin, leucoside, and gentisic acid 5-O-β-D-(6′-O-trans-4-coumaroyl)-glucopyranoside notably upregulated Ngb expression in the treated neuronal cells. It has also been reported that flavonoids such as formononetin, genistein, and daidzein increase Ngb promoter activity in both mouse and human neuronal cells [26]; thus, our findings also support the premise that natural extracts containing abundant levels of flavonoids and isoflavonoids exert their neuroprotective effects by increasing Ngb expression in target cells.

The present work assessed the neuroprotective properties of Ngb-mediated PCE toward ischemic injury in the tMCAO mouse model. In this study, ischemia-induced injury was documented and quantified by the extent of neurological deficits and infarct volume changes throughout the acute phase, and the PCE-treated tMCAO group was shown to have significantly improved damage resistance to the four types of neurological deficits and infarct volume changes. These results are consistent with previous findings that flavonoid-rich extracts and their flavonoid components promote the recovery of neurological function by lessening post-stroke neurological deficits and reducing infarct volume increases [43,44,45,46,47]. Our data point to neurobehavioral and cerebral infarction improvements along with increased levels of Ngb in the PCE-administered group. These findings in our cerebral ischemia model suggest that the neuroprotective properties of PCE may be due to the upregulation of Ngb levels by the flavonoids and isoflavonoids derived from PCE in the affected areas.

Ngb is well-characterized as a neuroprotectant that scavenges ROS against oxidative damage and protects neurons from apoptosis [11,12,13]. We expected that PCE, as an Ngb activator, would also have antioxidative and antiapoptotic properties. To demonstrate this, first, the antioxidant activity of PCE on intracellular levels of ROS levels was evaluated by oxidized DCFDA in vitro and in vivo. From oxidized DCFDA measurements, the application of PCE demonstrated consistent results for intracellular ROS inhibition in both primary cortical neurons and the ischemia-injured brain. 

To further evaluate the potential role of PCE in the antioxidant enzyme system, the GSH and CAT activities and SOD and NOX2 expression were measured in vitro and in vivo, respectively. The results indicate that the activities of CAT decreased in the presence of oxidative damage in N2a cells, but its activities increased with PCE treatment compared with those in the vehicle treatment. In the ischemic brain, we found that the expression of SOD in the PCE-treated tMCAO group was 1.5-fold higher than in the vehicle-treated tMCAO group. As a result of confirming NOX2 expression through gp91phox, the expression of NOX2 was increased in the ischemic area, whereas PCE treatment abolished NOX2 expression compared to the vehicle-treated tMCAO group. Taken together, our findings show that the antioxidant properties of PCE are also associated with increased SOD and CAT and decreased NOX2, suggesting that the antioxidant properties of PCE are manifested through the upregulation of the antioxidant enzyme system as well as Ngb.

Some experimental studies have reported that flavonoid-rich extracts exhibit antioxidant properties by inhibiting free radical production in oxidative stress-induced cells [48] and mitigating I/R-induced brain damage in rats [49]. It has also been shown that isoflavonoid-rich extracts exhibit potent antioxidant activity by scavenging ROS [50]. These previous studies agree with our observations, but further research is required to differentiate whether the antioxidant activity of PCE is due to the unique components of PCE or due to upregulated Ngb by PCE as a general source of isoflavonoids and flavonoids.

We next analyzed the effects of PCE application on oxidative signal changes using the activated p38 MAPK marker in the mouse samples. p38 MAPK is phosphorylated on both threonine 180 (Thr180) and tyrosine 182 (Tyr182) residues during oxidative stress [51], and according to our data, ischemic brains showed an apparent increase in the levels of activated p38 throughout the acute stroke phase in the core area after tMCAO, while PCE-treated mice had suppressed levels of activated p38 in both the core and penumbra areas. In addition to being a marker for enhanced ROS production, activated p38 is associated with increased apoptosis; thus, the changes in activated p38 levels presumably also point to PCE treatment exerting an antiapoptotic effect on the target tissues. 

Antiapoptotic activity has also been documented as being an additional function of increased Ngb levels [12]. In our system, in the core area after ischemic stroke, there was a negative correlation between changes in the expression of Ngb and levels of activated p38. In the samples analyzed, the levels of Ngb decreased in the core area, whereas those of p38 activation were increased. In comparison, PCE-treated mice had upregulated Ngb levels with a reduction in the levels of activated p38 in both the core and penumbra areas. For the apoptosis-related protein caspase-3, it was observed that PCE treatment suppressed its activation. The role of PCE in the reduction in cCasp3 was also confirmed in N2a cells exposed to oxidative damage, suggesting that the antiapoptotic activity of PCE is consistent both in vitro and in vivo. This observation supports the premise that the reduction in the levels of activated p38 points to the potent antiapoptotic action of PCE treatment in cerebral ischemia.

We also examined changes in the levels of survival-associated signals in the ischemic area. It has been reported that Ngb suppresses apoptosis by activating the PI3K/AKT pathway [52]. In our data, p-AKT expression, as a survival signal, appeared to increase together with increased Ngb expression upon PCE treatment. There was also rescue of prosurvival Bcl-2 levels in the PCE-treated group, as Bcl-2 levels decreased in the penumbra following cerebral ischemic stroke induction. The changes seen in these markers for apoptosis and survival support an antiapoptotic role for PCE treatment in the ischemic stroke mouse model.

According to our results, PC extract exhibits impressive antioxidative activity as an Ngb activator. However, further mechanistic studies will be necessary regarding the efficacy of PCE and its components. For instance, an analysis is required on whether the PCE induction of Ngb directly suppresses ROS production. In addition, a comparative analysis of the effectiveness of the extract (PCE) and compounds (PCCs) revealed that PCE (10^−7^–10^−5^ g/mL) increased Ngb expression and alleviated neurotoxicity. At a concentration of 10^−6^ M, active purified compounds such as prunetin, prunin, genistein, and gentisic acid 5-O-β-D-(6′-O-trans-4-coumaroyl)-glucopyranoside also increased Ngb expression and alleviated neurotoxicity. These in vitro comparisons indicate that the potency of PCE is as effective as that of purified compounds. To evaluate the in vivo effectiveness of PCE compared to PCCs, we are now examining the protective effects of prunetin and 5-O-β-D-(6′-O-trans-4-coumaroyl)-glucopyranoside in a mouse stroke model. Our efforts will provide the individual contributions of PCE-derived PCCs regarding the effectiveness and mechanism in cerebral ischemic stroke. Furthermore, a recent study demonstrated that a suboptimally active p38, defined by phosphorylation only on its Thr180, induces survival [53]. As such, there may be differential regulation of various phosphorylated p38 forms in the affected ischemic regions that need to be defined for the PCE mechanism of action. As PCE was discovered to act as a phytoestrogen in our previous study, the data in the present study were mainly obtained using female mice in an in vivo study. In a female stroke model, PCE significantly improved ischemic damage. On the other hand, PCE also improved cytotoxicity in mixed-sex primary neuron cultures, which confirmed the beneficial effects of PCE in both females and males. Our ongoing study showed that PCE also improved ischemic damage in the male stroke model. For further studies on sex-related mechanisms, it is also necessary to investigate the effect of PCE in separate-sex primary neuron culture [54]. A study on the proper dosing of PCE for optimal therapeutic efficacy in ischemic stroke is also required. Finally, these promising protective properties of PCE may need to be tested for additional cerebrovascular disease applications.

## 5. Conclusions

PCE was found to upregulate Ngb levels and to possess neuroprotective properties in a model of cerebral ischemia, as it suppressed stroke-induced ROS production and apoptosis. As a potent Ngb activator, our study suggests that PCE may be considered for therapeutic applications for cases that would benefit from increased Ngb levels, such as alleviating the functional damage from cerebral ischemia.

## Figures and Tables

**Table 1:** List of 18 compounds identified from PCE.

No.	Chemical Name	Structure	Molecular Formula (Molecular Weight)	No.	Chemical Name	Structure	Molecular Formula (Molecular Weight)
PCC1	*cis*-Melilotoside	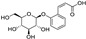	C_15_H_18_O_8_ (326.30)	PCC10	Sakuranetin	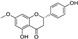	C_16_H_14_O_5_ (286.28)
PCC2	Genistin	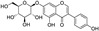	C_21_H_20_O_10_ (432.38)	PCC11	Prunin	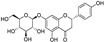	C_21_H_22_O_10_ (434.39)
PCC3	Genistein 5-glucoside	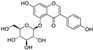	C_21_H_20_O_10_ (432.38)	PCC12	Epicatechin	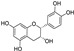	C_15_H_14_O_6_ (290.27)
PCC4	Prunetinoside	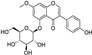	C_22_H_22_O_10_ (446.40)	PCC13	Quercetin 3-sophoroside	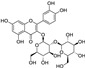	C_27_H_30_O_17_ (626.52)
PCC5	Genistein	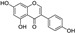	C_15_H_10_O_5_ (270.24)	PCC14	Quercetin 3-sambubioside	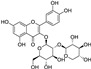	C_26_H_28_O_16_ (596.49)
PCC6	Prunetin	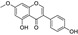	C_16_H_12_O_5_ (284.26)	PCC15	Sophoraflavonoloside	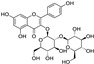	C_27_H_30_O_16_ (610.52)
PCC7	Naringenin	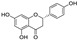	C_15_H_12_O_5_ (272.25)	PCC16	Hyperoside	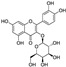	C_21_H_20_O_12_ (464.38)
PCC8	Sakuranin	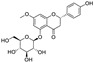	C_22_H_24_O_10_ (448.42)	PCC17	Leucoside	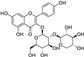	C_26_H_28_O_15_ (580.49)
PCC9	Neosakuranin	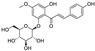	C_22_H_24_O_10_ (448.42)	PCC18	Gentisic acid 5-O-β-D-(6′-O-trans-4-coumaroyl)-glucopyranoside	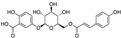	C_22_H_22_O_11_ (462.40)

**Table 2:** Neurological severity score (NSS) changes after PCE treatment in ischemic stroke mouse model. Evaluation of neurological deficits using four test parameters 1 day after ischemic stroke. The scores of the mice are marked according to the scales used. Values are mean ± SEM. ** *p* < 0.01, * *p* < 0.05, compared with the vehicle-treated tMCAO group, determined by Student’s *t*-test.

**Group**	**Number**	**Zea-longa**					**Mean ± SEM**
		**0**	**1**	**2**	**3**	**4**	
**Sham**	12	12	0	0	0	0	0
**tMCAO-Veh**	5	1	0	4	0	0	1.6 ± 0.40
**tMCAO-PCE**	7	2	1	4	0	0	1.3 ± 0.36
**Group**	**Number**	**Forelimb**					**Mean ± SEM**
		**0**	**1**	**2**	**3**	**4**	
**Sham**	12	12	0	0	0	0	0
**tMCAO-Veh**	5	0	1	3	1	0	2.0 ± 0.32
**tMCAO-PCE**	5	0	5	0	0	0	1 *
**Group**	**Number**	**Circling**					**Mean ± SEM**
		**0**	**1**	**2**	**3**	**4**	
**Sham**	12	12	0	0	0	0	0
**tMCAO-Veh**	4	1	0	3	0	0	1.5 ± 0.50
**tMCAO-PCE**	6	4	2	0	0	0	0.3 ± 0.21 *
**Group**	**Number**	**Prehensile traction**					**Mean ± SEM**
		**0**	**1**	**2**	**3**	**4**	
**Sham**	12	12	0	0	0	0	0
**tMCAO-Veh**	5	0	2	3	0	0	1.6 ± 0.24
**tMCAO-PCE**	7	5	2	0	0	0	0.3 ± 0.18 **

## Data Availability

The data presented in this study are available within the article.

## References

[1] Wardlaw J.M., Murray V., Berge E., Del Zoppo G.J. (2009). Thrombolysis for acute ischaemic stroke. Cochrane Database Syst. Rev..

[2] Eltzschig H.K., Eckle T. (2011). Ischemia and reperfusion–from mechanism to translation. Nat. Med..

[3] Andrabi S.S., Parvez S., Tabassum H. (2020). Ischemic stroke and mitochondria: Mechanisms and targets. Protoplasma.

[4] Chamorro A., Dirnagl U., Urra X., Planas A.M. (2016). Neuroprotection in acute stroke: Targeting excitotoxicity, oxidative and nitrosative stress, and inflammation. Lancet Neurol..

[5] Rama R., Rodríguez J. (2012). Excitotoxicity and Oxidative Stress in Acute Ischemic Stroke.

[6] Baron J.C. (2018). Protecting the ischaemic penumbra as an adjunct to thrombectomy for acute stroke. Nature reviews. Neurology.

[7] Burmester T., Weich B., Reinhardt S., Hankeln T. (2000). A vertebrate globin expressed in the brain. Nature.

[8] Guidolin D., Tortorella C., Marcoli M., Maura G., Agnati L.F. (2016). Neuroglobin, a Factor Playing for Nerve Cell Survival. Int. J. Mol. Sci..

[9] Dewilde S., Kiger L., Burmester T., Hankeln T., Baudin-Creuza V., Aerts T., Marden M.C., Caubergs R., Moens L. (2001). Biochemical characterization and ligand binding properties of neuroglobin, a novel member of the globin family. J. Biol. Chem..

[10] Trent J.T., Watts R.A., Hargrove M.S. (2001). Human neuroglobin, a hexacoordinate hemoglobin that reversibly binds oxygen. J. Biol. Chem..

[11] Lan W.B., Lin J.H., Chen X.W., Wu C.Y., Zhong G.X., Zhang L.Q., Lin W.P., Liu W.N., Li X., Lin J.L. (2014). Overexpressing neuroglobin improves functional recovery by inhibiting neuronal apoptosis after spinal cord injury. Brain Res..

[12] Raychaudhuri S., Skommer J., Henty K., Birch N., Brittain T. (2010). Neuroglobin protects nerve cells from apoptosis by inhibiting the intrinsic pathway of cell death. Apoptosis.

[13] Gao Y., Zhang Y., Dong Y., Wu X., Liu H. (2020). Dexmedetomidine Mediates Neuroglobin Up-Regulation and Alleviates the Hypoxia/Reoxygenation Injury by Inhibiting Neuronal Apoptosis in Developing Rats. Front. Pharmacol..

[14] Brittain T., Skommer J., Raychaudhuri S., Birch N. (2010). An antiapoptotic neuroprotective role for neuroglobin. Int. J. Mol. Sci..

[15] Narayan P., Manandhar S.M. (2002). Plants and People of Nepal.

[16] Joshi S.R. (2004). Himalayan cherry—Prunus cerasoides. Bee World.

[17] Jangwan J.S., Kumar N. (2015). Isolation and Characterization of New Flavonoid Glycoside from the Seeds of Prunus cerasoides. J. Med. Plants Stud..

[18] Bahuguna R.P., Jangwan J.S., Kaiya T., Sakakibara J. (1987). Puddumin-A, a New Flavanone Glucoside from Prunus cerasoides. J. Nat. Prod..

[19] Fatima A., Alok S., Agarwal P., Singh P.P., Verma A. (2013). Benefits of herbal extracts in cosmetics: A review. Int. J. Pharm. Sci. Res..

[20] Poonam V., Kumar G., Reddy L.C., Jain R., Sharma S.K., Prasad A.K., Parmar V.S. (2011). Chemical constituents of the genus Prunus and their medicinal properties. Curr. Med. Chem..

[21] Arora D.S., Mahajan H. (2018). In Vitro Evaluation and Statistical Optimization of Antimicrobial Activity of Prunus cerasoides Stem Bark. Appl. Biochem. Biotechnol..

[22] Sharma A., Joshi R., Kumar S., Sharma R., Rajneesh, Padwad Y., Gupta M. (2018). Prunus cerasoides fruit extract ameliorates inflammatory stress by modulation of iNOS pathway and Th1/Th2 immune homeostasis in activated murine macrophages and lymphocytes. Inflammopharmacology.

[23] Arora D.S., Mahajan H. (2019). Major Phytoconstituents of Prunus cerasoides Responsible for Antimicrobial and Antibiofilm Potential against Some Reference Strains of Pathogenic Bacteria and Clinical Isolates of MRSA. Appl. Biochem. Biotechnol..

[24] Sachdeva C., Kumar S., Kaushik N.K. (2021). Exploration of Anti-plasmodial Activity of Prunus cerasoides Buch.-Ham. ex D. Don (family: Rosaceae) and Its Wood Chromatographic Fractions. Acta Parasitol..

[25] Kim S.D., Kim Y., Kim M., Jeong H., Choi S.H., Ryu H.W., Oh S.R., Lee S.W., Li W.Y., Wu H.H. (2020). Estrogenic properties of Prunus cerasoides extract and its constituents in MCF-7 cell and evaluation in estrogen-deprived rodent models. Phytother. Res..

[26] Liu N., Yu Z., Gao X., Song Y.S., Yuan J., Xun Y., Wang T., Yan F., Yuan S., Zhang J. (2016). Establishment of Cell-Based Neuroglobin Promoter Reporter Assay for Neuroprotective Compounds Screening. CNS Neurol. Disord. Drug Targets.

[27] Liu N., Yu Z., Xiang S., Zhao S., Tjarnlund-Wolf A., Xing C., Zhang J., Wang X. (2012). Transcriptional regulation mechanisms of hypoxia-induced neuroglobin gene expression. Biochem. J..

[28] Kim M., Kim S.D., Kim K.I., Jeon E.H., Kim M.G., Lim Y.R., Lkhagva-Yondon E., Oh Y., Na K., Chung Y.C. (2021). Dynamics of T Lymphocyte between the Periphery and the Brain from the Acute to the Chronic Phase Following Ischemic Stroke in Mice. Exp. Neurobiol..

[29] Cao Y., Sun N., Yang J.W., Zheng Y., Zhu W., Zhang Z.H., Wang X.R., Shi G.X., Liu C.Z. (2017). Does acupuncture ameliorate motor impairment after stroke? An assessment using the CatWalk gait system. Neurochem. Int..

[30] Yang J., Ahn H.N., Chang M., Narasimhan P., Chan P.H., Song Y.S. (2013). Complement component 3 inhibition by an antioxidant is neuroprotective after cerebral ischemia and reperfusion in mice. J. Neurochem..

[31] Combs D.J., D’Alecy L.G. (1987). Motor performance in rats exposed to severe forebrain ischemia: Effect of fasting and 1,3-butanediol. Stroke.

[32] Jeong J., Kim S., Lim D.S., Kim S.H., Doh H., Kim S.D., Song Y.S. (2017). TLR5 Activation through NF-kappaB Is a Neuroprotective Mechanism of Postconditioning after Cerebral Ischemia in Mice. Exp. Neurobiol..

[33] Shen F., Jiang L., Han F., Degos V., Chen S., Su H. (2019). Increased Inflammatory Response in Old Mice is Associated with More Severe Neuronal Injury at the Acute Stage of Ischemic Stroke. Aging Dis..

[34] Sanchez-Bezanilla S., Nilsson M., Walker F.R., Ong L.K. (2019). Can We Use 2,3,5-Triphenyltetrazolium Chloride-Stained Brain Slices for Other Purposes? The Application of Western Blotting. Front. Mol. Neurosci..

[35] Yu Z., Liu N., Li Y., Xu J., Wang X. (2013). Neuroglobin overexpression inhibits oxygen-glucose deprivation-induced mitochondrial permeability transition pore opening in primary cultured mouse cortical neurons. Neurobiol. Dis..

[36] Herold S., Fago A., Weber R.E., Dewilde S., Moens L. (2004). Reactivity studies of the Fe(III) and Fe(II)NO forms of human neuroglobin reveal a potential role against oxidative stress. J. Biol. Chem..

[37] Song N., Ma J., Meng X.W., Liu H., Wang H., Song S.Y., Chen Q.C., Liu H.Y., Zhang J., Peng K. (2020). Heat Shock Protein 70 Protects the Heart from Ischemia/Reperfusion Injury through Inhibition of p38 MAPK Signaling. Oxid. Med. Cell Longev..

[38] Manabat C., Han B.H., Wendland M., Derugin N., Fox C.K., Choi J., Holtzman D.M., Ferriero D.M., Vexler Z.S. (2003). Reperfusion differentially induces caspase-3 activation in ischemic core and penumbra after stroke in immature brain. Stroke.

[39] Guo C., Yang M., Jing L., Wang J., Yu Y., Li Y., Duan J., Zhou X., Li Y., Sun Z. (2016). Amorphous silica nanoparticles trigger vascular endothelial cell injury through apoptosis and autophagy via reactive oxygen species-mediated MAPK/Bcl-2 and PI3K/Akt/mTOR signaling. Int. J. Nanomed..

[40] Randolph S.A. (2016). Ischemic Stroke. Workplace Health Saf..

[41] Zivin J.A. (2009). Acute stroke therapy with tissue plasminogen activator (tPA) since it was approved by the U.S. Food and Drug Administration (FDA). Ann. Neurol..

[42] Ciccone L., Nencetti S., Socci S., Orlandini E. (2021). Neuroglobin and neuroprotection: The role of natural and synthetic compounds in neuroglobin pharmacological induction. Neural. Regen. Res..

[43] He Q., Li S., Li L., Hu F., Weng N., Fan X., Kuang S. (2018). Total Flavonoids in Caragana (TFC) Promotes Angiogenesis and Enhances Cerebral Perfusion in a Rat Model of Ischemic Stroke. Front. Neurosci..

[44] Luo Y., Cui H.-X., Jia A., Jia S.-S., Yuan K. (2018). The Protective Effect of the Total Flavonoids of Abelmoschus esculentus L. Flowers on Transient Cerebral Ischemia-Reperfusion Injury Is due to Activation of the Nrf2-ARE Pathway. Oxidative Med. Cell. Longev..

[45] Tu X., Wang M., Liu Y., Zhao W., Ren X., Li Y., Liu H., Gu Z., Jia H., Liu J. (2019). Pretreatment of Grape Seed Proanthocyanidin Extract Exerts Neuroprotective Effect in Murine Model of Neonatal Hypoxic-ischemic Brain Injury by Its Antiapoptotic Property. Cell Mol. Neurobiol..

[46] Liang W., Huang X., Chen W. (2017). The Effects of Baicalin and Baicalein on Cerebral Ischemia: A Review. Aging Dis..

[47] Tang Z., Li M., Zhang X., Hou W. (2016). Dietary flavonoid intake and the risk of stroke: A dose-response meta-analysis of prospective cohort studies. BMJ Open.

[48] Cirmi S., Maugeri A., Lombardo G.E., Russo C., Musumeci L., Gangemi S., Calapai G., Barreca D., Navarra M. (2021). A Flavonoid-Rich Extract of Mandarin Juice Counteracts 6-OHDA-Induced Oxidative Stress in SH-SY5Y Cells and Modulates Parkinson-Related Genes. Antioxidants.

[49] Zhang S., Qi Y., Xu Y., Han X., Peng J., Liu K., Sun C.K. (2013). Protective effect of flavonoid-rich extract from Rosa laevigata Michx on cerebral ischemia-reperfusion injury through suppression of apoptosis and inflammation. Neurochem. Int..

[50] Chiang H.M., Chiu H.H., Liao S.T., Chen Y.T., Chang H.C., Wen K.C. (2013). Isoflavonoid-Rich Flemingia macrophylla Extract Attenuates UVB-Induced Skin Damage by Scavenging Reactive Oxygen Species and Inhibiting MAP Kinase and MMP Expression. Evid. Based Complement Altern. Med..

[51] Cuadrado A., Nebreda A.R. (2010). Mechanisms and functions of p38 MAPK signalling. Biochem. J..

[52] Zhang B., Liu Y., Li Y., Zhe X., Zhang S., Zhang L. (2018). Neuroglobin promotes the proliferation and suppresses the apoptosis of glioma cells by activating the PI3K/AKT pathway. Mol. Med. Rep..

[53] Martinez-Limon A., Joaquin M., Caballero M., Posas F., de Nadal E. (2020). The p38 Pathway: From Biology to Cancer Therapy. Int. J. Mol. Sci..

[54] Fairbanks S.L., Young J.M., Nelson J.W., Davis C.M., Koerner I.P., Alkayed N.J. (2012). Mechanism of the sex difference in neuronal ischemic cell death. Neuroscience.

